# Supplementary material for: Molecular analysis of aggressive renal cell carcinoma with unclassified histology reveals distinct subsets
Source: Nat Commun. 2016 Oct 7;7:13131. doi: 10.1038/ncomms13131 (PMC5059781; doi:10.1038/ncomms13131)
Supplement: Supplementary Information — Supplementary Figures 1-9 and Supplementary Tables 1-2 [file ncomms13131-s1.pdf]

Supplementary Figures

a

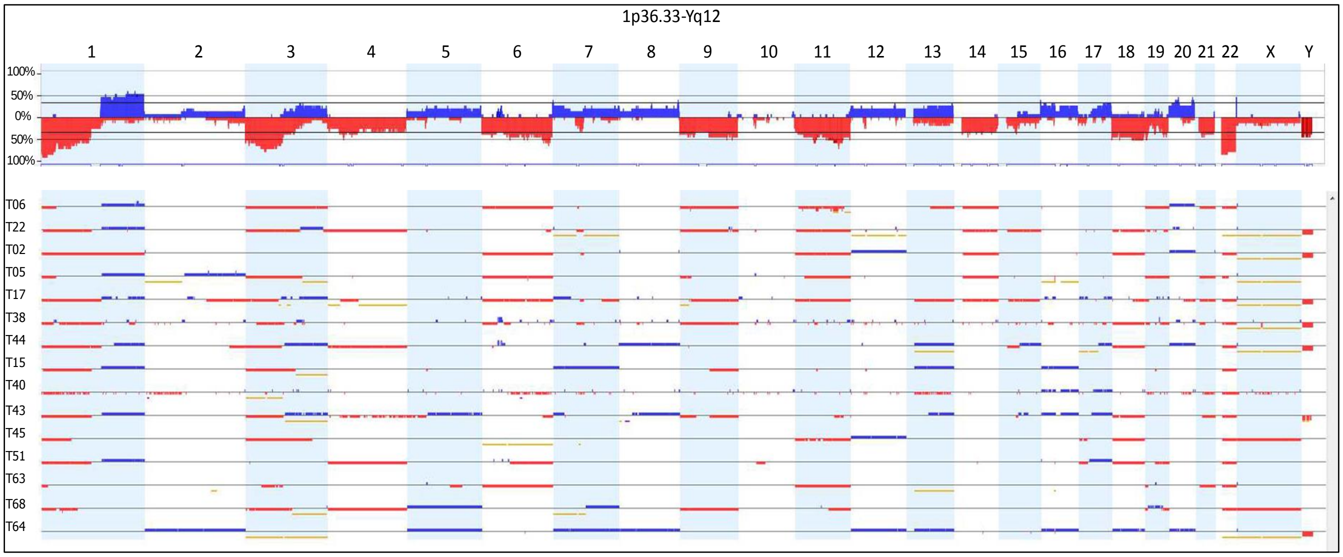

b

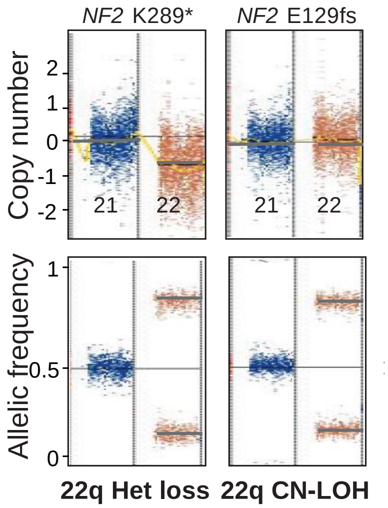

Supplementary Figure 1. Copy number (CN) changes and allelic imbalances (AI) detected by OnsoScan SNP-array analysis in 15 uRCC samples with *NF2* mutation or 22q loss. (**a**) Heat map of SNP-array results including CN gains (blue), CN losses (red), and copy neutral loss of heterozygosity (CN-LOH, yellow) are displayed for each individual case (rows) with chromosomes organized in columns and indicated by labels on the top. Arm-level unbalanced genomic alterations in 1p (loss), 3p (loss and CN-LOH), and 22q (loss and CN-LOH) occurred at frequencies of more than 50% in this subset of uRCC tumors. (**b**) Representative cases with concurrent *NF2* mutation and 22q hemizygous loss or 22q CN-LOH as demonstrated by the copy number view (top) and allelic frequency view (bottom).

a

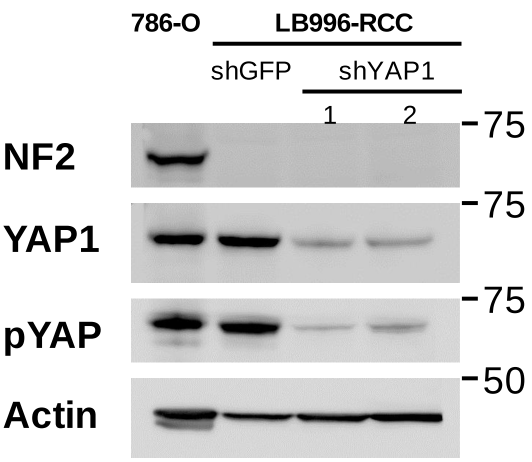

b

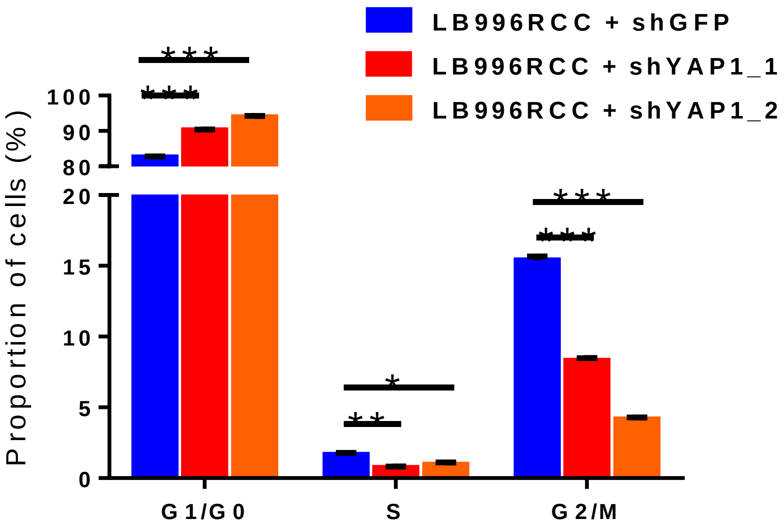

Supplementary Figure 2. Knockdown of YAP1 in LB996-RCC cells results in a decrease of proliferating cells in S or G2/M phase. (**a**) Immunoblots with the indicated antibodies (left) in LB996-RCC cells with YAP1 or control (GFP) knockdown are shown. 786-O is a control RCC cell line. (**b**) Results of cell cycle analysis in LB996-RCC cells with YAP1 or control (GFP) knockdown are shown in bar graph. Bars, mean values; error bars, S.E.M .; replicates n=3. Statistical significance was determined by Student's t-test. Statistical significance is indicated as *** $p<0.001$; ** $p<0.01$; * $p<0.05$.

a

b

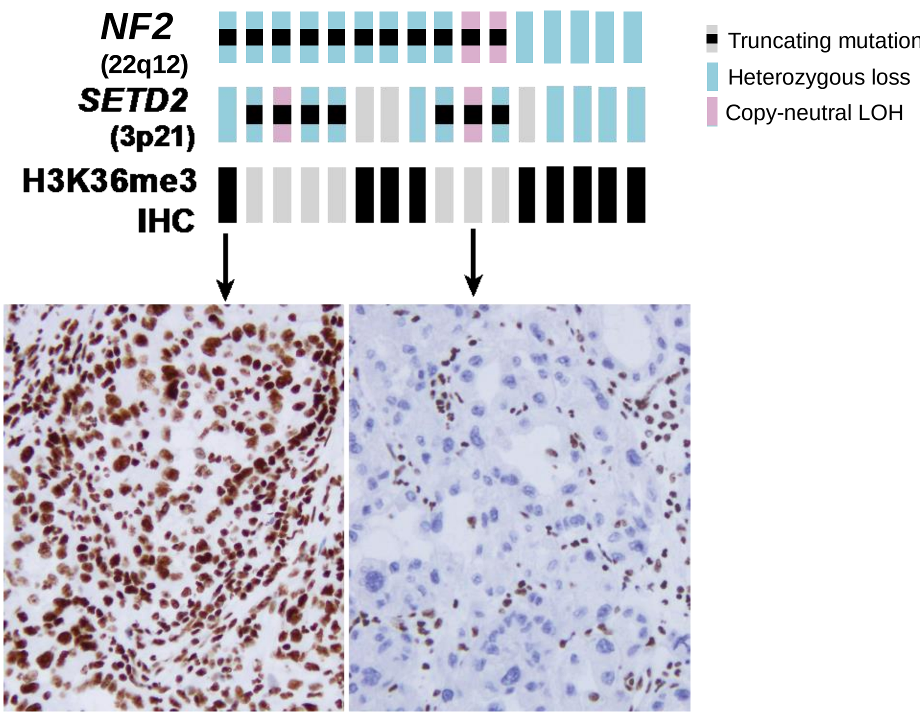

Supplementary Figure 3. *SETD2* mutations are enriched in NF2-loss subset and correlate with the loss of H3K36me3 histone mark. (**a**) Schematic overview of the *NF2* and *SETD2* mutations, copy number loss at their corresponding genomic loci, and result of histone H3K36me3 immunohistochemistry. (**b**) Representative images of uRCC tumors with either retained expression of H3K36me3 mark (left) or a loss of this histone mark (right). Note that endothelial, inflammatory or stromal cells serve as internal positive control.

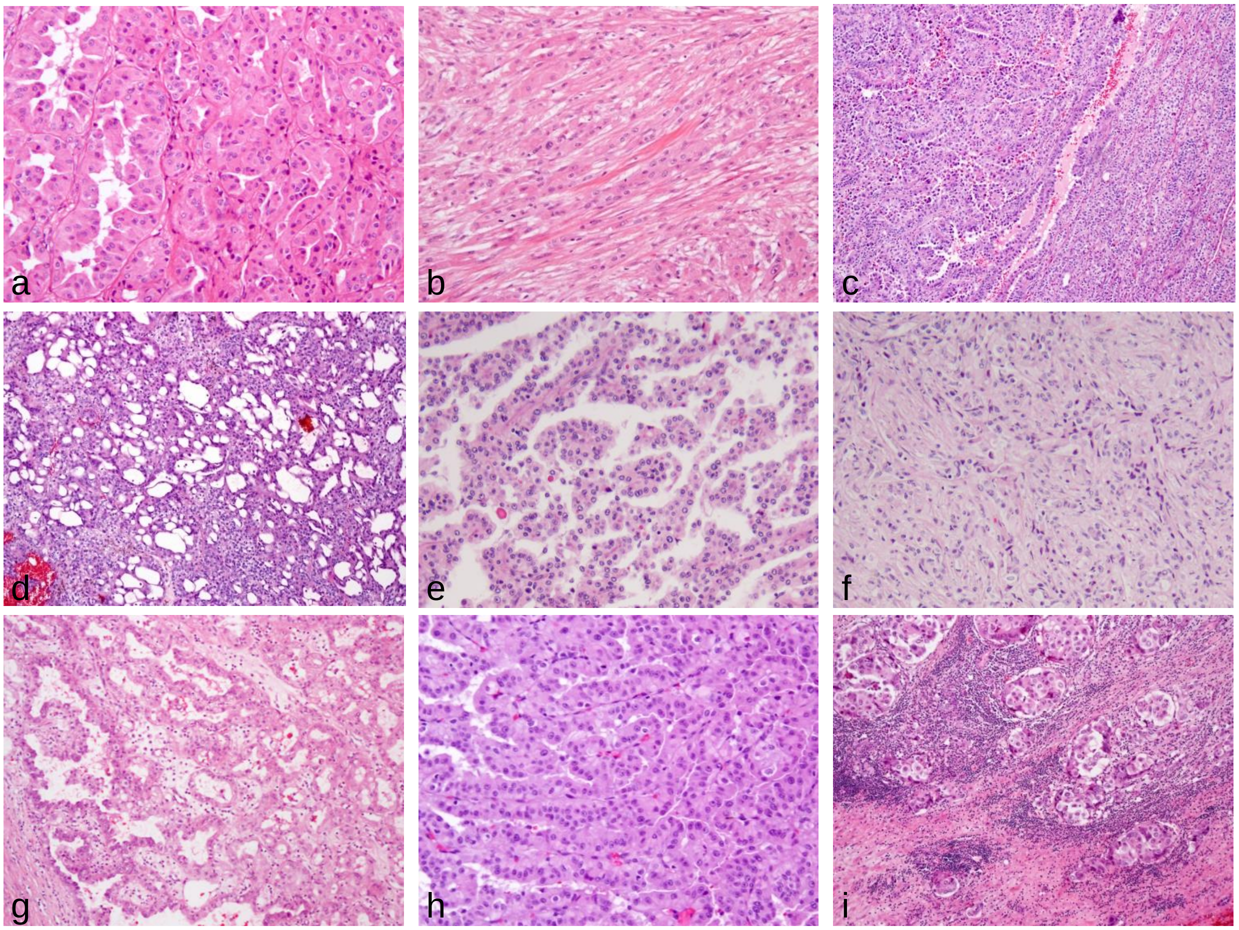

Supplementary Figure 4. The NF2-loss uRCC often exhibits complex architectures with multinodular or infiltrative growth. T15 showed tubular, tubulopapillary and sarcomatoid patterns (**a-b**); T02 had papillary, solid, and microcystic/cribriform areas (**c-d**); T45 showed micropapillary, papillary, solid and sarcomatoid patterns (**e-f**); T22 exhibited mixed tubulopapillary and tubulocystic areas (**g**); T05 and T43 showed focal papillary architecture (**h**) or collecting duct carcinoma-like area (**i**), respectively.

a

ID	HLRCC nuclear Features	2SC IHC	FH IHC	*FH* Germline Alteration	*FH* Somatic Alteration	Comment
T35	Yes	Pos	Heterogeneous (lost/weakly retained)	c.568delAC (p.T190fs)	p.234_235insT	HLRCC
T39	Yes	Pos	lost	c.1431insAAA (p.477_478insK)<br>c.683T>A (p.I228N)	LOH<br>LOH	HLRCC
T42	Yes	Pos	lost	c.1189G>A (p.G397R)	p.S419*	HLRCC
T41	Yes	Pos	lost	Not tested	Homozygous deletion	FH-deficiency (somatic)
T71	No	Neg	Retained	Not tested	p.G401V	Passenger mutation

b

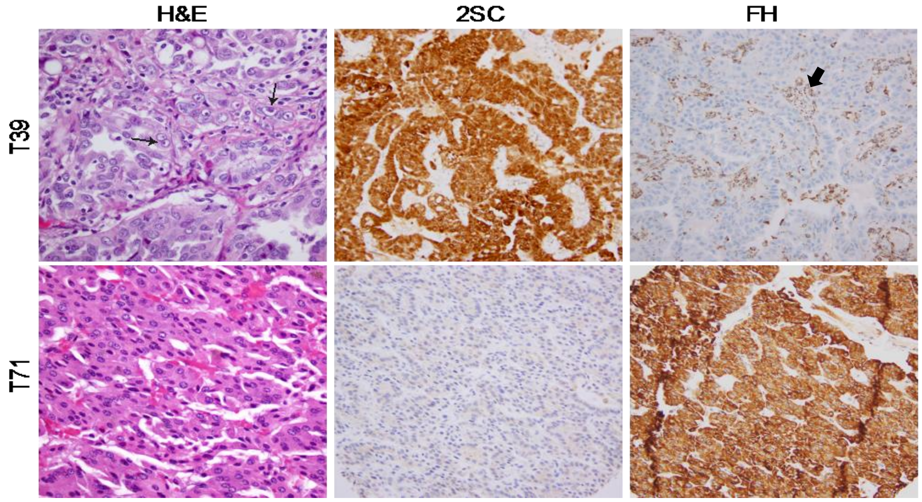

c

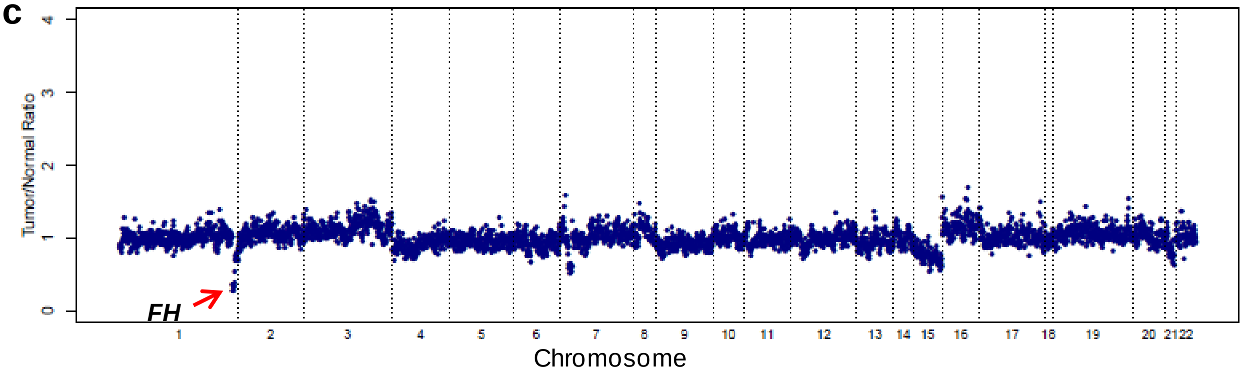

Supplementary Figure 5. FH alterations detected in the uRCC cohort. (**a**) Summary table of 5 uRCC tumors either with *FH* mutation/copy number abnormalities or exhibiting immunohistochemistry (IHC) pattern (2SC positive, FH negative/lost) consistent with FH-deficiency. (**b**) Representative histologic, 2SC and FH immunostaining images of T39 and T71. Arrows, characteristic nuclear features seen in HLRCC cases; block arrow, internal control of retained FH staining in endothelial, inflammatory and stromal cells. (**c**) Copy number blot derived from IMPACT sequencing data indicated a somatic homozygous loss of *FH* gene in T41.

a

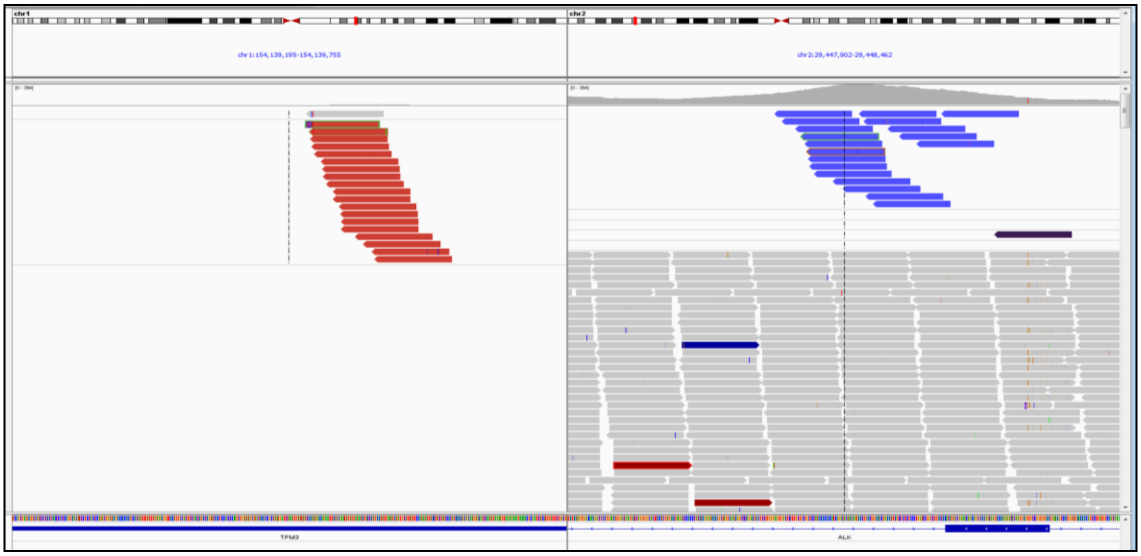

b

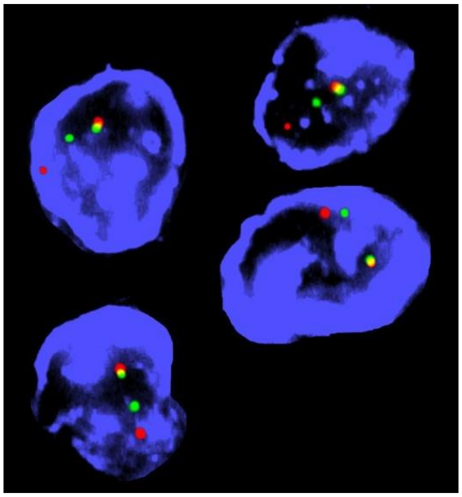

Supplementary Figure 6. TPM3-ALK fusion detected in uRCC cohort. (**a**) Integrated Genomic Viewer (IGV) snapshot of T12 illustrates the breakpoint in *TPM3* on chromosome 1 (left, red) and the breakpoint in *ALK* on chromosome 2 (right, blue). (**b**) ALK break-apart FISH assay showed a separation of red and green signals in one allele, confirming the presence of ALK translocation.

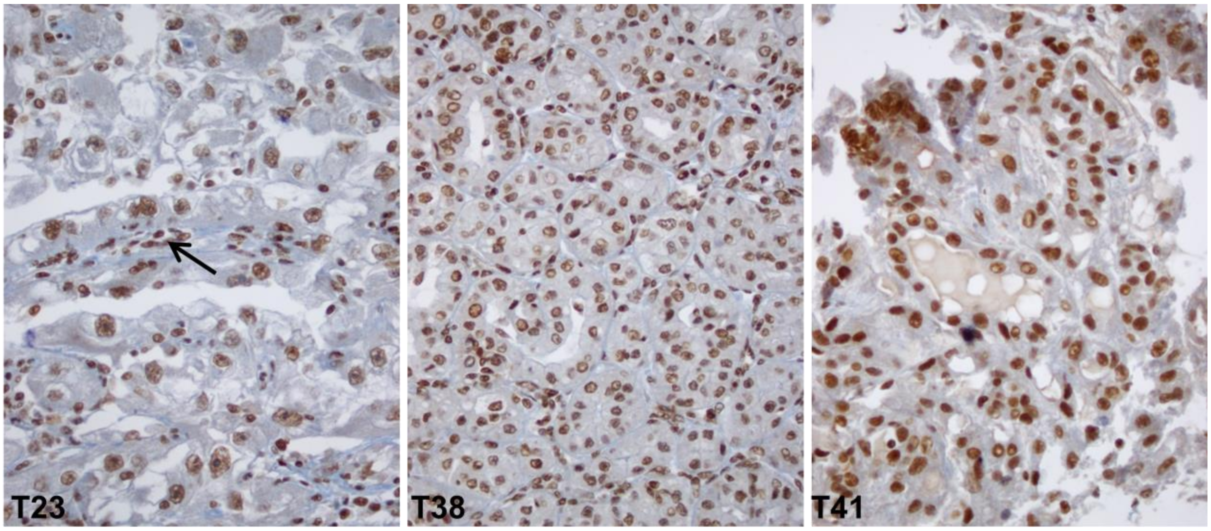

Supplementary Figure 7. INI1 immunohistochemistry in uRCC tumors with *SMARCB1* somatic mutation. Three tumors with *SMARCB1* splice site or missense mutations (T23, T38, and T41) did not exhibit INI1 protein loss. Note that the endothelial, inflammatory and stromal cells (arrow) served as internal positive controls.

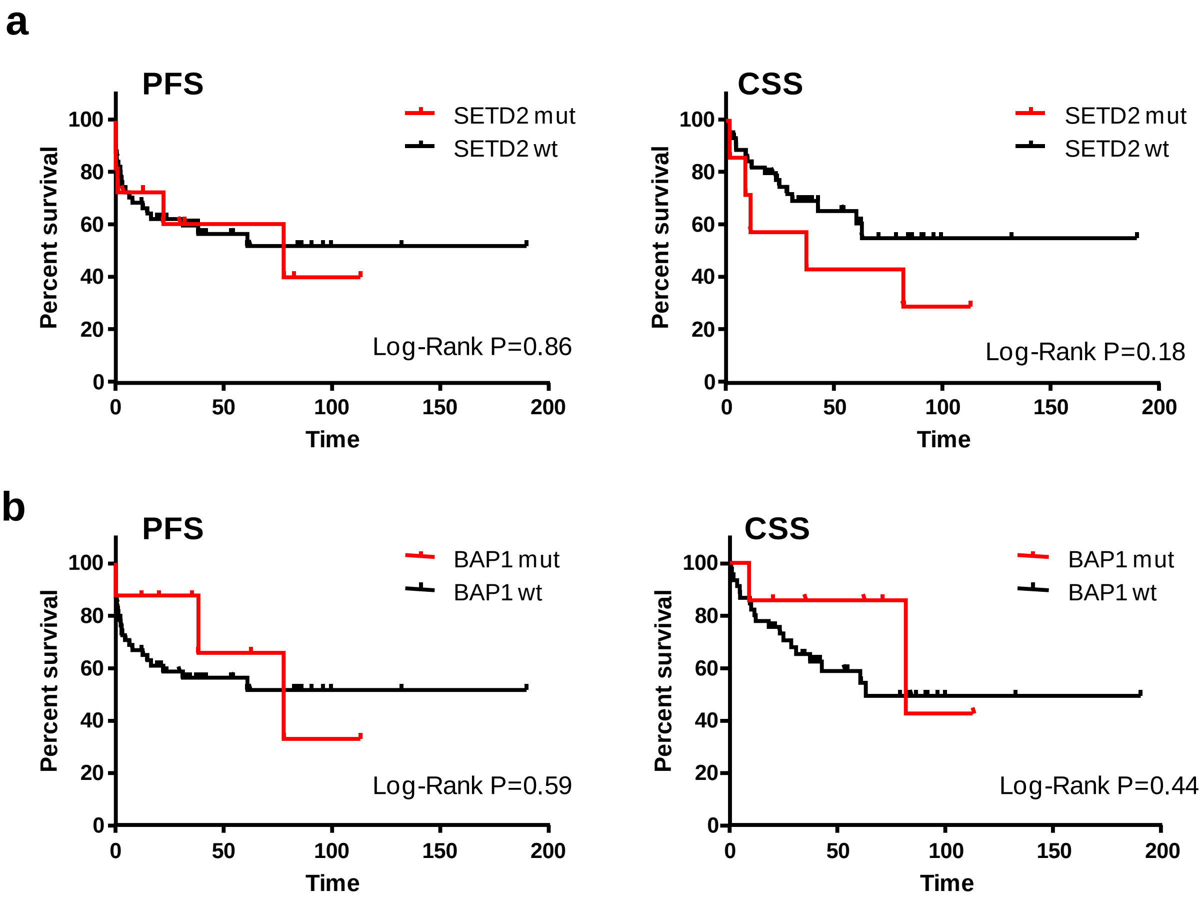

Supplementary Figure 8. Survival analysis for recurrent somatic alterations in uRCC cohort. The Kaplan-Meier survival plots for progression-free survival (PFS) and cancer-specific survival (CSS) are shown for SETD2 (**a**) or BAP1 (**b**) wild type (wt) and mutant (mut) cases. p values were calculated using Log-Rank test.

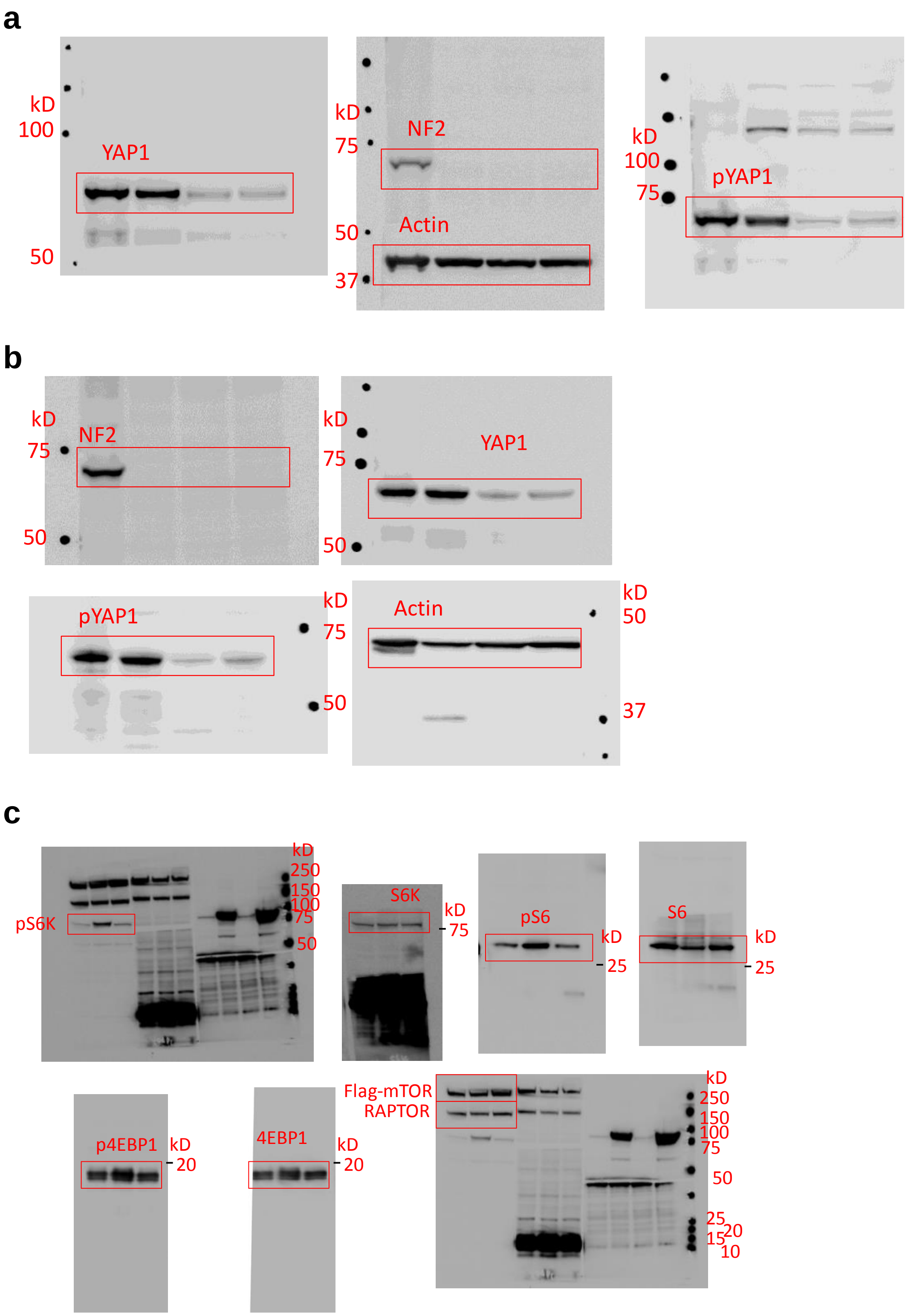

Supplementary Figure 9. Full blots of protein blot analyses conducted in the study. (**a**) ACHN cells with YAP1 or control (GFP) knockdown in Fig. 2g. (**b**) LB996-RCC cells with YAP1 or control (GFP) knockdown in Supplementary Fig. 2. (**c**) 293T cells transfected with the Flag-tagged MTOR expression constructs in conjunction with HA-S6K in Fig. 3c.

Supplementary Table 1. Patient characteristics and outcomes in a cohort of high-grade unclassified RCC (uRCC) (n=62)

Age , median (range) (at nephrectomy)	60 (12-86)
Gender	
Male	34 (55%)
Female	28 (45%)
Median tumor size-cm (range)	6.0 (1.0-18.0)
T stage (at nephrectomy)	
pT1	22 (36%)
pT2	4 (6%)
pT3/T4	36 (58%)
Regional lymph node (at nephrectomy)	
N0/Nx	42 (68%)
N1	20 (32%)
Distant metastases (at last follow-up)	
M0	36 (58%)
M1	26 (42%)
Median time to distant met-month (range)	10.2 (1-77.6)
Overall 5-year survival	56.9%
Median survival-month (95% CI)	65.6 (42.6-107.9)
RCC-specific mortality	22 (35%)
Median follow-up time in survivors-month	53.6

Supplementary Table 2. Three-color probes used in NF2/22q FISH analysis				
**Probe**	**Locus**	**Gene / STS Marker**	**Label**	**Clone**
Probe 1:	22q12.2	*NF2*	Red	RP11-155B12, RP1-76B20
Probe 2:	22q11.1	D22S43 (control)	Orange	RP11-701M12, RP11-1037C4
Probe 3:	10q10	Cen10 (control for ploidy)	Green	p10RP8
